# Gender Equality Trends of First Authors in Publications of Artificial Intelligence and Thyroid

**DOI:** 10.7759/cureus.45820

**Published:** 2023-09-23

**Authors:** Susmitha Devi Chalamalasetti, Silbin Tamrakar, Preyansh Doshi, Neera N Vora, Vishnu Karrothu, Abhinav Reddy Pathe

**Affiliations:** 1 Obstetrics and Gynecology, Kakatiya Medical College, Warangal, IND; 2 Internal Medicine, Enam Medical College and Hospital, Dhaka, BGD; 3 Internal Medicine, Gujarat Cancer Society (GCS) Medical College, Hospital and Research Centre, Ahmedabad, IND; 4 Internal Medicine, All India Institute of Medical Sciences, Bhopal, Bhopal, IND; 5 General Practice, Davao Medical School Foundation Inc., Davao City, PHL

**Keywords:** pubmed, first author, gender equality, thyroid, artificial intelligence

## Abstract

Thyroid diseases are diverse, ranging from benign conditions to potentially life-threatening disorders. Recently, the application of artificial intelligence (AI) in evaluating thyroid disease has significantly enhanced medical research, diagnosis prediction, and algorithm development. Coupled with this advancement is the rising focus on the importance of gender equality in scientific publications. This study delves into the gender trends of first authors in papers related to “Artificial Intelligence and Thyroid” sourced from PubMed from 2003 to 2022, scrutinizing these trends based on both country and year.

A bibliometric analysis was conducted on PubMed to retrieve relevant articles over this 19-year time span. Following this, the names and affiliated countries of the first authors were determined. The Namsor app, a tool for classifying personal names by gender, origin, or ethnicity, was then used to segregate the data based on gender. Statistical analyses were performed using the R software- ARIMA model and Fisher’s exact test was applied to examine the correlations between gender and country of origin.

From the 254 analyzed articles, 43.5% of the first authors were female, while 56.69% were male. The year 2022 saw the most significant number of female first-author publications. Intriguingly, the European Journal of Radiology was prominent due to its favorable gender ratio. Moreover, the association between gender and country was significant, with China being a standout.

Limitations included focusing only on PubMed journals and using a third party for gender identification. Nevertheless, the study reveals a move toward gender parity in AI and thyroid research over the past 18 years, emphasizing the importance of sustained efforts for academic inclusivity.

## Introduction and background

Research and development in the context of artificial intelligence (AI) uses machines to simulate the activities of human or human-like brains. Image identification, supplementary diagnosis, medicine research, and health management are just a few of the areas of medical treatment where AI has made a significant impact [1].

After years of training, doctors can finally learn how to diagnose, treat, and perform treatments. Similar to humans, machines may be trained to accomplish certain tasks with enough information. In conventional computer programming, the output is produced by hand-coding precise instructions. AI can, therefore, map the relationship between input and output variables using the available data. For instance, if a system receives ultrasound images of thyroid nodules that have been classified as benign or cancerous, AI algorithms can forecast the diagnosis if we provide the algorithm with a fresh, unseen image of a thyroid nodule. Artificial neural network technology advancements, faster computing systems, and graphics processing units have all helped AI in medicine perform better in recent years. However, even with these developments, molecular markers are one of the only uses of AI-driven solutions in thyroid disorders [2].

To establish the place of AI in thyroid disorders, multicenter, multi-device, well-conducted, prospective trials are required. The use of AI-driven tools can help interpreters of thyroid nodules and thyroid cytopathology images make more objective decisions. Increased use of AI in thyroid disorders has the potential to reduce the need for thyroid operations, thyroid nodule biopsies, and overall spending on healthcare [2].

Academic publications help in career development. Gender inequalities in publication can mark gender inequalities in grants and scholarships for conducting research, the establishment of labs, publication support and funding, and on the clinical front. Therefore, gender trends are important to establish differences that may be due to disparities in the field of advanced medicine. Advanced access and better gender equality may be the reasons for differences among countries.

This study aims to analyze the gender trends of first authors in publications related to “Artificial Intelligence and Thyroid” from PubMed-indexed publications from 2003 to 2022 based on country and year; to analyze the trends in gender representation of first authors over the 19-year study period and forecast future trends; and to determine any affiliations of the first authors and explore any associations between gender trends and countries.

## Review

This study is a bibliometric analysis that was conducted on June 27, 2023. PubMed was used to search for the articles related to “Artificial Intelligence and Thyroid” using the Boolean operation “Artificial Intelligence and Thyroid” [title]. Articles from the last 20 years, i.e., from January 1, 2003, to December 31, 2022, were included in the study. Publications from 2023 were excluded, as only a few publications were available. Articles accepted in the year 2022 and published and appearing on PubMed in 2023 were included in the study. All search results were then imported into Microsoft Excel for further analysis.

The total number of entries, i.e., 254, was equally divided among all six authors. Then, using the title of the article/DOI on PubMed, we found the full name of the author and determined their country based on their affiliated institution. Using these details, Namsor, which is an Application Programming Interface (API), was used to determine the gender of the first author based on the name and the country. Namsor is a name-checking software that classifies personal names by gender, country of origin, or ethnicity [3].

Fisher’s exact test was used to determine if there was any association between gender trends and country. Gender trends were analyzed and future trends were also determined. Statistical analysis was done using R software, the ARIMA model, and graphs were prepared using DataWrapper.

A total of 254 articles were eligible for inclusion in the study. Among these, 110 (43.3%) of the first authors were identified as female, while 144 (56.69%) were identified as male, resulting in a female-to-male ratio of the first authors of 0.76.

**Figure 1:**
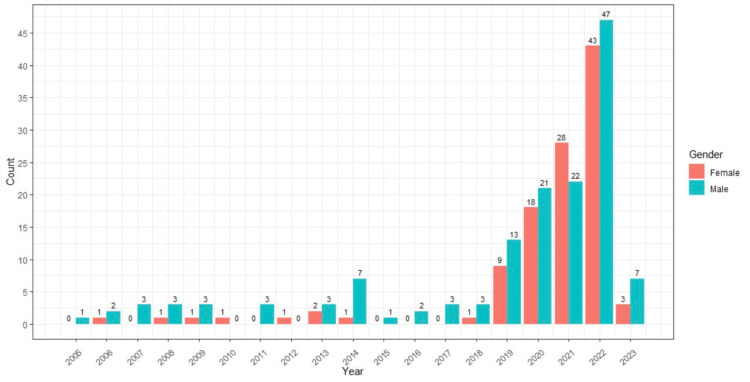
The annual distribution of male and female first authors from 2005 to 2023.

Figure 1 displays the annual distribution of male and female first authors from 2005 to 2023. The highest number of female first-author publications in the field of “Artificial Intelligence and Thyroid” was observed in 2022, with a total of 43 publications.

**Figure 2:**
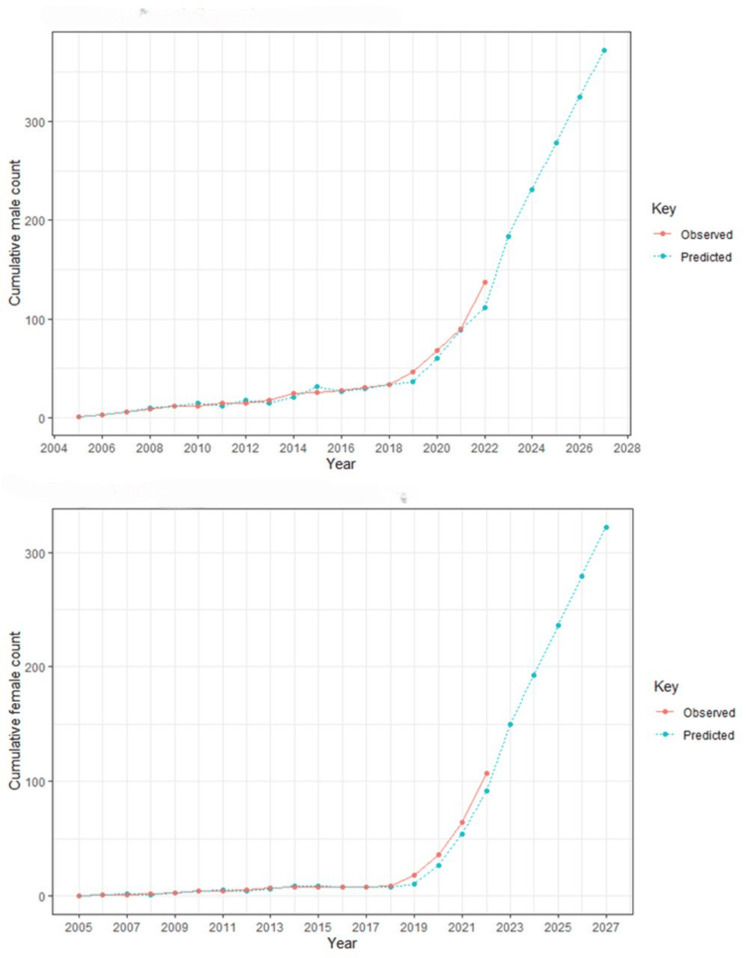
The publication trends of cumulative male and female first authors from 2005 to 2022, along with predictions for the next five years.

Figure 2a illustrates the publication trends of cumulative male first authors from 2005 to 2022, along with predictions for the next five years, and Figure 2b showcases the publication trends of cumulative female first authors from 2005 to 2022 forecasting future trends for the next five years. According to the ARIMA model used in this analysis, it is expected that by the year 2027, there will be approximately 370 publications by male first authors and around 330 publications by female first authors. The data used for modeling ranges from 2005 to 2022, as 2023 data is incomplete.

**Figure 3:**
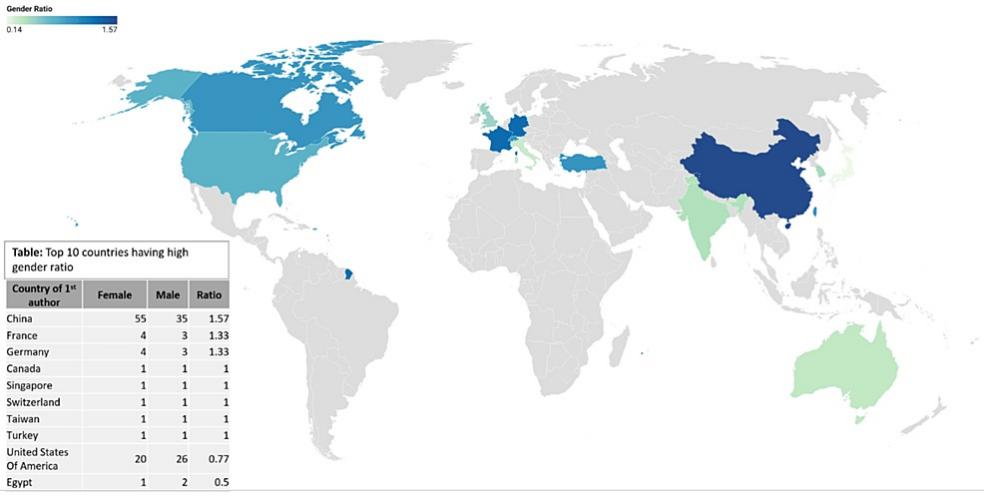
The gender trends in publications from 2005 to 2023 categorized by the country of origin.

Figure 3 displays the gender trends in publications from 2005 to 2023 categorized by the country of origin. Among the countries examined, China exhibits the highest female-to-male gender ratio of first authors with a ratio of 1.57, followed by France and Germany with ratios of 1.33. Conversely, Egypt has the least favorable female-to-male gender ratio of first authors at 0.5, followed by the United States with a ratio of 0.77.

Table 1 showcases the top 10 journals with favorable gender ratios. Remarkably, the European Journal of Radiology stands out with the highest favorable gender ratio, while the least favorable gender ratio is observed in the Frontiers of Oncology.

**Table 1:** Top journals having a favorable gender ratio.

Journal/Book	Female	Male	Ratio
Frontiers of Oncology	4	5	0.8
European Journal of Radiology	4	3	1.33
Journal of Pathology Informatics	2	2	1
Thyroid	2	2	1
Annals of Surgical Surg Oncology	1	1	1
Artificial Intelligence in Medicine Med	1	1	1
Biomedicines	1	1	1
Cancer	1	1	1
Chinese Medical Journal (Engl)	1	1	1
Computational Intelligence and Neuroscience	1	1	1

Table 2 specifically focuses on the top 10 journals with a higher gender ratio. It is noteworthy that the European Journal of Radiology has the highest gender ratio with a ratio of 1.33.

**Table 2:** Top journals having a high gender ratio.

Journal/Book	Female	Male	Ratio
European Journal of Radiology	4	3	1.33
Journal of Pathology Informatics Inform	2	2	1
Thyroid	2	2	1
Annals of Surgical Oncology	1	1	1
Artificial Intelligence in Medicine Med	1	1	1
Biomedicines	1	1	1
Cancer	1	1	1
Chinese Medical Journal (Engl)	1	1	1
Computational Intelligence and Neuroscience	1	1	1
Cytopathology	1	1	1

This study aims to report information on gender equality among authors in thyroid disorders related specifically to AI. In our study of 254 articles, 43.3% had females as first authors from the year 2005 to 2023 [4]. The year 2022 marked the highest number of females as first publishers. Based on future projections, it is anticipated that by the year 2027, there will be a notable increase in the number of papers published with females as first authors, surpassing 300 publications.

A closer look at data across countries reveals varying gender ratios in authorship. Impressively, China leads with a ratio of 1.57 females to every male author. France is not far behind at 1.33, whereas the United States trails with a ratio of 0.77. In stark contrast, the Republic of Korea has the lowest representation of females with a ratio of 0.44. Drawing from OECD data, we note that France’s high ratio may be influenced by its 44% female doctors compared to the United States’s 34% and Korea’s 22% [5]. This trend suggests a positive correlation: countries with more female doctors seem to produce a higher number of female-led research publications.

The past decade has witnessed a commendable rise in the representation of women in academic medicine. Given this trend, it is unsurprising to observe a correlating increase in articles from more recent years featuring women as the primary authors [4]. This upward trajectory is mirrored in various studies across medical disciplines. For instance, a study from PubMed highlighted that in psychiatry journals between 2008 and 2018, female authorship rose from 40.0% in 2008 to 44.8% by 2018 [6]. Similarly, a 2019-2020 article from the Journal of the American Academy of Dermatology showcased that 51% of matched dermatology applicants were female authors [7]. The British Medical Journal also reported on the evolving gender trends in oncology research, noting a jump from 26.6% female authors in 2002 to 32.9% in 2019 [8]. Further emphasizing this pattern, a Journal of Nephrology publication revealed that first authorship by women in major US nephrology journals increased from 32% in 2011 to 40% in 2019 [9].

However, some areas still lag. A striking contrast is the endocrinology field, where a 70-year analysis recorded a female authorship of only around 22.2%, notably lower than our study’s findings [4]. Similarly, the Journal of Endocrinology, Metabolism and Diabetes of South Africa reported a modest 28.9% female authorship over 25 years [10].

Exploring other areas of medicine, Bluth et al.’s 35-year examination into radiology authorship signaled positive gender parity trends, with a surge in articles featuring female senior authors [11]. Similarly, Ruble et al. highlighted the shifting paradigms in gender development research, emphasizing broader perspectives in understanding gender dynamics [12]. Interestingly, the recent global pandemic’s repercussions on gender disparities in research were underscored by Son et al., indicating the potential exacerbation of existing inequalities during such crises [13].

Overall, while some advancements hint at promising changes, such as the findings from Filardo et al.’s 10-year analysis [14], achieving consistent gender equality in research remains a work in progress. The collective findings emphasize the ongoing gender disparities in research and authorship but also spotlight areas witnessing positive change.

It is crucial to continue efforts to bridge the gender gap by addressing structural barriers, biases, and other factors that contribute to gender disparities. Promoting gender equality in research encourages diverse perspectives, improves the quality of scientific knowledge, and fosters a more inclusive research community.

Our study encountered some limitations. First, we focused solely on PubMed-indexed journals. Further studies can include other search engines and applications with higher accuracy. Second, only the first author data was included and the author gender was generated by a third-party website (Namsor). This approach introduces the potential for inaccuracies. Third, we were unable to obtain the full names of certain first authors, resulting in their omission from the analysis. Finally, we only analyzed gender representation as the first author in PubMed. However, we have no reason to expect that the excluded journals would differ substantially from the analyzed journals. Additionally, gender ratios may not be the best measure of gender equality, as it does not account for variations in authorship roles (e.g., first author vs. senior author) or even the quality and impact of research and publication.

## Conclusions

Over the past 18 years, there has been a notable increase in achieving gender parity in the field of AI and thyroid. This progress indicates positive strides toward fostering gender equity in the field. The findings of this study offer valuable insights that can inform future initiatives and interventions aimed at addressing gender disparities in academia. By leveraging the potential of women in leadership positions, these efforts can contribute to fostering a more equitable and diverse culture within the field of research.
